# Symptomatic Morgagni's hernia in an elderly patient

**DOI:** 10.4103/0974-2700.58654

**Published:** 2010

**Authors:** Santosh PV Rai, Madhav Kamath, Ashok Shetty, Suresh Shenoy, Ashvini Kumar

**Affiliations:** Department of Radiodiagnosis, KMC Hospital, Attavar, Mangalore - 575 001, Karnataka, India; 1 Department of Thoracic Surgery, KMC Hospital, Attavar, Mangalore - 575 001, Karnataka, India; 2 Department of Gastroenterology, KMC Hospital, Attavar, Mangalore - 575 001, Karnataka, India

**Keywords:** Hernia, Morgagni, obstruction

## Abstract

Hernia of Morgagni occurs through an anterior defect in the diaphragm. Symptoms of these hernias are attributable to the herniated viscera. In our case, there was partial obstruction due to herniation of the distal stomach and pylorus into the right hemithorax that was reduced surgically through a right thoracolapaorotomy. Of special emphasis are the various modalities used to diagnose this condition in our case.

## INTRODUCTION

Hernia through the foramen of Morgagni (HM) is uncommon in adults, accounting for only 3% of all treated diaphragmatic hernias.[1] Hernia of Morgagni occurs through an anterior defect in the diaphragm but usually presents in adulthood through a paramedian defect. In contrast, it occurs in a central retrosternal location in children.[2] It could be an incidental diagnosis in adulthood or can present with obstructing symptoms of the herniated viscera.[3] Symptoms of these hernias are attributable to the herniated viscera. Morgagni hernias containing bowel may require repair on presentation because of the risk of incarceration.[4]

## CASE REPORT

**Figure 1:**
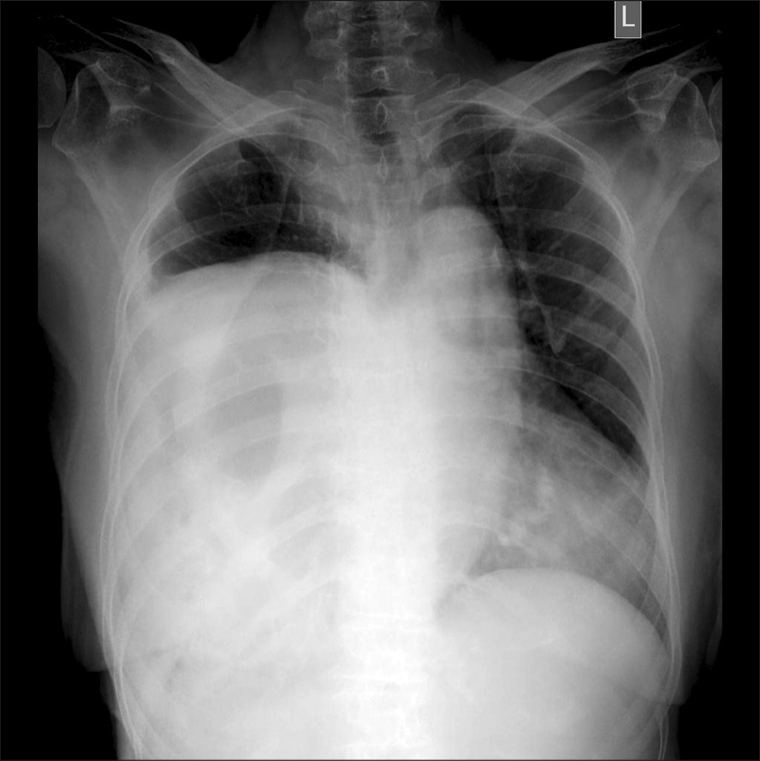
Plain radiograph showed opacification of the entire lower part of the right hemithorax with air lucencies within

**Figure 2:**
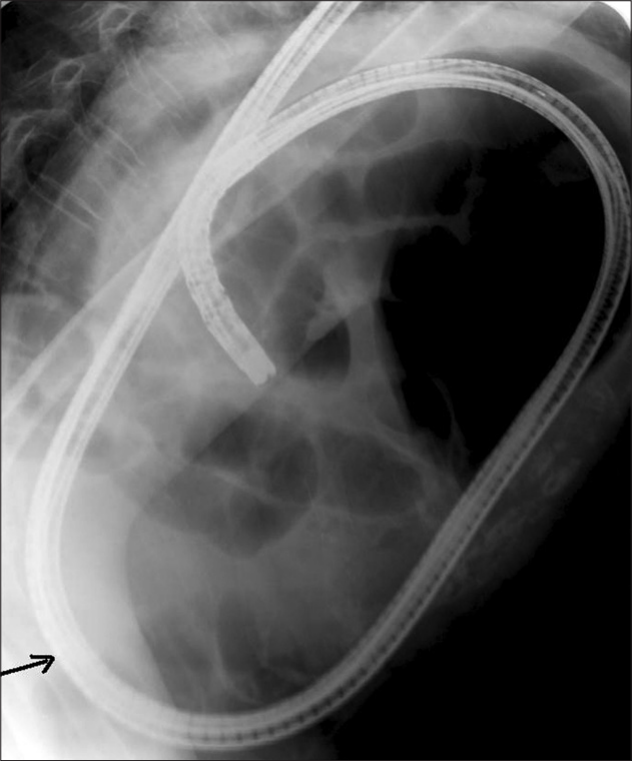
Lateral radiograph of the chest taken during endoscopy shows the scope to enter the air-fluid level within the right hemithorax (black arrow), suggesting right diaphragmatic hernia

**Figure 3:**
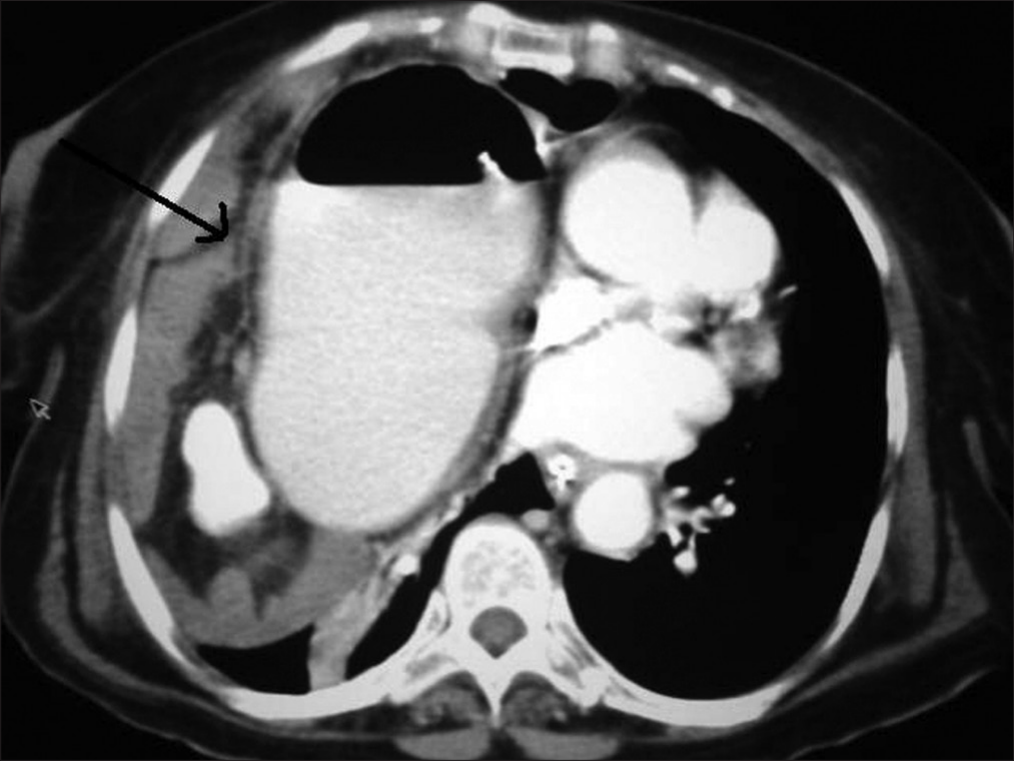
Computed tomography scan confirmed the diaphragmatic hernia with herniation of the stomach, pylorus, ascending and transverse colon into the right hemithorax (black arrow)

**Figure 4:**
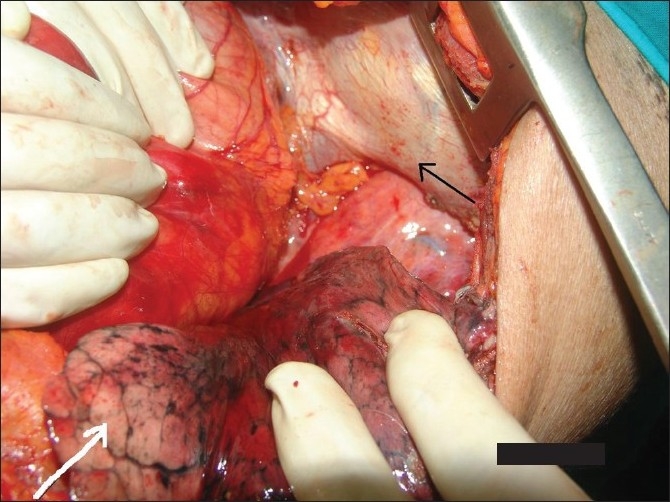
The herniated contents were noted in the right hemithorax with a collapsed right lung (white arrow, lung; thick black arrow, diaphragm)

A 72-year-old woman presented to emergency with recurrent abdominal pain and vomiting from 4 months and acute abdomen for 1 week. She complained of non-bilious, non-projectile vomiting containing food particles. Visible peristalsis was appreciated, which appeared aggravated after taking food. She did not complain of any history of trauma or breathlessness. On clinical examination, her vitals were stable as she had been resuscitated in emergency and she was evaluated with radiographs, endoscopy and computed tomographic (CT) examinations. Plain radiograph performed on the day of admission, 1 week from the onset of acute symptoms, showed opacification of the entire lower part of the right hemithorax with significant air lucency within [Figure 1]. The opacification was silhouetting the right cardiac border, confirming its anterior location. The cardia and mediastinum were shifted to the left. The appearance of air lucencies within the lesion confirmed the presence of the bowel in the right hemithorax, which confirmed the diagnosis of diaphgrammatic hernia. Because the patient had also complained of hematemesis in the last week, endoscopy was performed on the same morning, which showed the esophagus filled with fluid residue. The gastric lumen appeared twisted at around 40 cm from the incisor level. The scope was negotiated beyond this point with mild difficulty and a lateral radiograph of the chest was taken. It showed the scope to enter the air-fluid level within the right hemithorax [Figure 2], suggesting right diaphragmatic hernia and herniation of the stomach into the right hemithorax. CT scan was performed with oral contrast, rectal contrast and intravenous contrast immediately on the day of admission. CT scan showed herniation of the stomach, pylorus, ascending and transverse colon with the fatty omentum in the right hemithorax [Figure 3]. The oral contrast had predominantly accumulated in the bowel loop in the right hemithorax with very little contrast in the distal small bowel. The transverse colon opacified with rectal contrast was also noted in the right hemithorax on the CT scan of the chest and upper abdomen. On surgery, a small defect between the sternal and costal fibers of the diaphragm was noted in a paramedian location. The herniated contents were noted in the right hemithorax with a partially collapsed right lung [Figure 4]. The volvulus of the stomach that was suspected pre-operatively was absent. The gastric outlet obstruction was due to the herniation of the distal stomach and pylorus into the narrow defect. Because there were adhesions to be released, a right thoracolaporotomy was performed. The hernia was reduced after releasing the adhesions and the diaphragmatic defect was repaired.

## DISCUSSION

Diaphragmatic hernia of Morgagni-Larrey is a rare entity that usually presents on the right side. These hernias occur in the anterior midline/paramedian location through the sternocostal hiatus of the diaphragm called the foramen of Morgagni. They are usually discovered incidentally or when they become symptomatic due to intestinal involvement (occlusive symptoms) or when respiratory dysfunction occurs.[5,6]

In a review of the literature, Rodriquez *et al*.[7] and Iso *et al*.[8] have described similar series of cases of symptomatic hernias due to intestinal obstruction that presented in elderly patients.

They are usually asymptomatic and often found incidentally on chest radiography. Routine radiographic studies are usually sufficient to arrive at the diagnosis, but a CT scan and sonography may be necessary. Surgery is appropriate for the management of symptomatic adult patients with Morgagni hernias, particularly those with findings of intestinal strangulation, with laparoscopic treatment an alternative approach in selected cases.[4] Few symptomatic hernias have been reported in the literature with and without gastric volvulus. Sometimes, the transverse colon can herniate through the defect and cause intestinal obstruction.[8] In our case, there was partial obstruction due to herniation of the distal stomach and pylorus, which was reduced surgically through a right thoracolapaorotomy.

## CONCLUSIONS

This case report highlights the unusual presentation of obstructed viscera within a Morgagni's hernia in a senile and elderly patient. Of special emphasis are the various modalities used to diagnose this condition. Although uncommon, herniation of the omentum and the bowel can occur in the adult through the foramen of Morgagni. This report highlights a case scenario where an elderly patient may require emergency intervention on presentation because of the risk of strangulation of the bowel.
